# Pair Matcher (*PaM*): fast model-based optimization of treatment/case-control matches

**DOI:** 10.1093/bioinformatics/bty946

**Published:** 2018-11-16

**Authors:** Eran Elhaik, Desmond M Ryan

**Affiliations:** 1 Department of Animal and Plant Sciences, University of Sheffield, Sheffield UK, UK; 2 INSIGNEO Institute for In Silico Medicine, University of Sheffield, Sheffield UK, UK

## Abstract

**Motivation:**

In clinical trials, individuals are matched using demographic criteria, paired and then randomly assigned to treatment and control groups to determine a drug’s efficacy. A chief cause for the irreproducibility of results across pilot to Phase-III trials is population stratification bias caused by the uneven distribution of ancestries in the treatment and control groups.

**Results:**

Pair Matcher (*PaM*) addresses stratification bias by optimizing pairing assignments *a priori* and/or *a posteriori* to the trial using both genetic and demographic criteria. Using simulated and real datasets, we show that *PaM* identifies ideal and near-ideal pairs that are more genetically homogeneous than those identified based on competing methods, including the commonly used principal component analysis (PCA). Homogenizing the treatment (or case) and control groups can be expected to improve the accuracy and reproducibility of the trial or genetic study. *PaM’s* ancestral inferences also allow characterizing responders and developing a precision medicine approach to treatment.

**Availability and implementation:**

*PaM* is freely available via *R*https://github.com/eelhaik/PAM and a web-interface at http://elhaik-matcher.sheffield.ac.uk/ElhaikLab/.

**Supplementary information:**

Supplementary data are available at *Bioinformatics* online.

## 1 Introduction

It is well recognized that pharmaceutical research and development (R&D) is in crisis. The number of new drugs approved per billion US dollars spent on R&D has halved roughly every 9 years since 1950 (Scannell *et al*., 2012) as spending in the industry has inflated to an average of ∼$5.8 billion per drug in 2011 compared to $1.3 billion per drug in 2005 (Roy, 2012). The latter phases of clinical trials test the drug’s efficacy compared to a placebo or other treatments in a randomized trial setting and require assessing tens, hundreds (Phase II trials) and eventually tens of thousands (Phase-III trials) of volunteers over a long period of time to prove that there is substantial evidence of a clinical benefit of the drug. Only 1 in 12 drugs that enters human clinical trials ends up gaining approval from the Food and Drug Administration. It is acknowledged that one of the biggest drivers of the increase in R&D costs is the regulatory process governing Phase-III clinical trials of new pharmaceuticals (Roy, 2012). As the regulatory environment is unlikely to relax (Scannell *et al*., 2012), it is important to understand why randomized control trials may be more successful in smaller trials.

Matching treatment (or case) with control groups is the most elementary and critical part of any trial (or study). Mismatched groups introduce genetic heterogeneity that may obscure performance of the trialled drug, e.g. due to genetic pre-disposition to response to the treatment, and result in reduced reproducibility between different cohorts (Scannell *et al*., 2012). Currently, individuals are matched based on demographic criteria (e.g. age, gender and self-reported ‘race’) and then randomly assigned to treatment and controls groups. It is well acknowledged that due to the significant heterogeneity among humans, demographic-based matching alone is inadequate. Trials are, thereby, vulnerable to ‘stratification bias,’ i.e. differences in genetic ancestry between individuals, which are not factored in when trial participants are grouped based on demographics alone. This undetected bias may contribute to biased interpretation of trial results due to lack of genetic information that may confound interpretation, leading to alterations in the false negative or false positive results, with subsequent financial and patient health consequences (Supplementary Fig. S1). In large groups, the stratification bias may be less pronounced, however, it is practically unavoidable in the case of rare diseases due to the difficulties in recruiting genetically homogeneous participants (Yusuf and Wittes, 2016). Crucially, this bias is more severe in small cohorts, leading to an applied misinterpretation of the drug’s efficacy that will be difficult to replicate in larger trials.

Population stratification can be addressed by optimizing the treatment-control matches *a priori* or/and *a posteriori* to the trial using a variety of tools applied to the genotype data and selecting matched pairs for downstream analyses. Due to the historically high cost of genotyping and sequencing, *a priori* methods rely heavily on demographic-based matching criteria followed by statistical corrections made *a posteriori*, if at all. *A priori* methods have long been considered biased, inaccurate and unhelpful (De Bono, 1996; Fustinoni and Biller, 2000; McAuley *et al.*, 1996) due to their reliance on self-reported ‘race’ (‘Africans’, ‘Asians’ and ‘European-Americans’ or ‘Whites’) or regional similarity, which does not eliminate the bias (Campbell *et al*., 2005; Chikhi *et al*., 2010; Elhaik *et al*., 2014; Wang *et al.*, 2006; Yusuf and Wittes, 2016). Unable to completely account for choices made at the *a priori* stage, *a posteriori* methods may make over-simplified, unrealistic or problematic assumptions (Kimmel *et al*., 2007), particularly concerning population structure. Computing the principal components (PCs) of the genotype matrix and adjusting the genotype vectors by their projections on the PCs is a popular method of accounting for population structure (Price *et al*., 2006). However, linear projections cannot be assumed to sufficiently correct for the effect of stratification due to other unaccounted confounders (Kimmel *et al*., 2007). PCs also ignore the complexity of population structure, are influenced by uneven sampling, and cannot properly represent individuals of mixed origins (Elhaik *et al*., 2014; Lacour *et al*., 2015; McVean, 2009; Yang *et al.*, 2012;). Even newer tools (Epstein *et al.*, 2007; Kimmel *et al*., 2007; Lacour *et al*., 2015) can make only basic assumptions concerning population structure and may ignore admixture or demographic criteria.

We developed Pair Matcher (*PaM*)—a genetic-based tool that optimizes pairing assignments *a priori* and/or *a posteriori* to the trial. *PaM* matches samples by demographic and genetic criteria and allows trial designers to make informed decisions in real time (Supplementary Fig. S1). *PaM* models individual genomes as consisting of gene pools (or admixture components) that correspond to their recent demographic history (Das *et al.*, 2016; Elhaik *et al*., 2014). *PaM* then matches individuals based on their age, gender and the similarity of their admixture components. We first compared the accuracy of *PaM_simple_* and *PaM_full_* and then the accuracy of the best performing tool to pairings made either at random, based on racial-criteria, or through PC analysis (PCA). Finally, we compared *PaM*’s pairing accuracy to those of clustering tools used in population genetic and genome-wide association studies (GWAS) in analyzing unmixed and mixed individuals. We also assessed reproducibility.

Optimizing the trial design can be expected to homogenize the treatment (or case) and control pairs and improve the accuracy and reproducibility of the trial or genetic study. This can be expected to lower drug developmental costs and benefit patients. Together with biogeographical tools that can predict the geographical origins of the responders (Elhaik *et al*., 2014), *PaM* can also be used to guide precision medicine approaches to treatment, for instance, in characterizing a subgroup of responders or mutation carriers (Baughn *et al*., 2018) and designing follow up trials focussing on this group.

## 2 Materials and methods

### 2.1 Simulated population datasets

We generated 24 datasets composed of 980–1000 individuals each in ADMIXTURE’s Q file format (individuals × proportion of admixture components) (Alexander *et al.*, 2009). Here and throughout this work, we adopted the admixture model of Elhaik *et al.* (2014) of nine admixture components representing: *North East Asia*, the *Mediterranean*, *South Africa*, *South West Asia, Native America*, *Oceania*, *South East Asia*, *Northern Europe* and *Sub-Saharan Africa.* Each dataset consisted of a file with nine admixture components generated randomly for individuals and their matching pairs and normalized so that each row would sum to 1. Dataset 1 consisted of 500 identical pairs (Supplementary Fig. S2). The genetic heterogeneity between the pairs of Datasets 2–8 was increased in a controlled manner by modifying the admixture components of one individual from each pair of Dataset 1 through an increasing perturbation of *X* [0…20%] subtracted from the odd numbered admixture components and added to the even numbered admixture components. The perturbation percentage was applied alternately (negative to the first component, positive to second component etc.) to prevent normalization to reverse the perturbation.

To assess pairing in more imperfect datasets, the remaining datasets were created by removing random individuals from the original datasets. Datasets 9–16 were created by removing one individual from each cohort (*remove-1*) and Datasets 17–24 were created by removing 20 individuals from each cohort (*remove-20*), leaving datasets of 999 and 980 individuals, respectively.

### 2.2 Worldwide population dataset

**Fig. 1.**
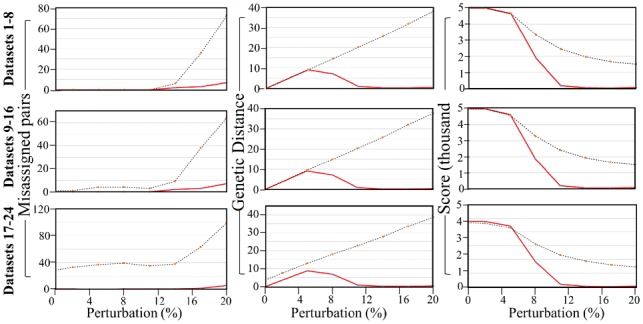
*PaM_simple_* performances on simulated datasets. Rows show the results of eight perturbed datasets [full dataset (left), *remove-1* (centre) and *remove-20* (right)]. *PaM_simple_* was applied without a threshold (dashed) and with a threshold of 7 (solid red). Columns show the number of individuals assigned to a different pair than their original counterpart per dataset (*x*-axis), total GD between all matched pairs and total score (maximum of 10 per pair with the three datasets having *n*_1_ = 500, *n*_2_ = 499, and *n*_3_ = 480 pairs, respectively)

We used the Genographic dataset, which comprises of ∼128 000 markers genotyped in 633 unrelated worldwide individuals of known geographic origins who have four grandparents from their population affiliation and geographic region of origin (Elhaik *et al*., 2014). We created a database of 13 mixed individuals of distinct ancestries by hybridizing 13 Indians with 13 British to yield a final cohort of 646 individuals. The hybridization was done by merging an even amount of random single nucleotide polymorphisms (SNPs) from random Indian and British individuals and calculating the admixture components of these genomes (Elhaik *et al*., 2014). The admixture components of the Genographic and simulated individuals (Elhaik *et al*., 2014) (Fig. 1) were provided as input to *PaM.* We also analyzed 40 Bedouin and 40 Pakistani (25 Brahui and 15 Burusho) individuals (Patterson *et al*., 2012) and calculated their nine admixture components as in Elhaik *et al.* (2014).

### 2.3 Unmixed and mixed population datasets

We used the Lazaridis *et al.* (2014) dataset that comprises of ∼600 841 markers genotyped in 2345 unrelated worldwide individuals. From each population that had at least four individuals, we selected two pairs of individuals who showed the highest identity-by-state (IBS) similarity to each other as inferred by PLINK (—cluster—matrix) (Purcell *et al*., 2007). From the 42 populations (168 individuals) identified in that manner, 100 random individuals were paired to a random member of their population, creating the unmixed dataset (*n* = 200). A mixed dataset was next created by randomly selecting three individuals and using the consecutive thirds of their genomes to create a 3 × hybrid individual. A matching pair was created in a similar way using different individuals from the same populations. This process was repeated 100 times (*n* = 200). Similarly, we created 5× and 7× datasets of the same size. Finally, we assembled three combined datasets that consist of: unmixed +3× mixed (*n* = 400), the latter dataset +5× mixed (*n* = 600) and the latter dataset +7× mixed (*n* = 800).

### 2.4 Comparing *PaM*_simple_ and *PaM*_full_

To optimize matches, *PaM* analyzes the age (optional), gender (optional) and admixture components for each individual in the studied cohort. These three parameters are obtained from PLINK’s fam file [using columns 4 (age) and 6 (gender)] and ADMIXTURE’s *Q* file. The Genetic Distance (GD) between the paired individual is defined as $\sqrt{{\sum^{\;}}_{k}{\left(i_{k}-j_{k}\right)}^{2}}$, where *i* and *j* are the individuals with *k* admixture components. *PaM* calculates the *nxn* GD matrix for each possible pairing, where *n* is the number of individuals in the *Q* file. Each element of the matrix, specified by row *i* and column *j*, corresponds to a pair (*i*, *j*). The matrix is symmetric with respect to the diagonal, which contains all zeros. A corresponding *nxn* score matrix is calculated as follows: pairs that are age (within 5 years by default) and gender matched get one point. Nine additional points are awarded for every matching admixture component if |*i_k_–j_k_*| ≤ 1% for the pair (*i*, *j*). An ideally matched pair has a score of 10 (age/gender and nine admixture components). An optimal pairing solution for Dataset 1 that consists of 500 pairs would be a GD of 0 between all pairs, a total score of 5000 (top score of 10 for 500 pairs), and no unpaired individuals (Supplementary Fig. S3).

*PaM* operates in two modes: *PaM_simple_* and *PaM_full_.* The *PaM_simple_* algorithm starts by selecting matrix row 1 (individual 1) and finding the column *j* which yields the minimum GD for pair (1, *j*). This matrix element corresponds to the first pair with row index 1 and column index *j.* Row 1 and column *j* and their symmetric element (*j*, 1) are removed from the GD matrix. Row 2 is next selected (provided it has not been removed in the previous step) and the column which yields the minimum GD is selected to form the second pairing. The corresponding rows and columns are then removed from the matrix. The optimization proceeds until all possible pairings are created and all unpaired individuals are stored. If the test cohort is an odd number then at least one unpaired individual is expected. The paired and unpaired individuals are reported in separate text files. To filter pairings with a score lower than a specified acceptable value, a user-controlled threshold was implemented.

The threshold is related to the expected genetic homogeneity of the pairs. A high threshold would result in homogeneous pairs and a large number of unpaired individuals. A threshold of 7 indicates that the pair’s age and gender matched as well as six of their admixture components.

When matrix rows have multiple identical minima, there is a potential dilemma since the specific row minimum selection could affect subsequent pairings (due to the row/column removal upon pair selection), and the final pairing solution may not be optimal. We explored different selection schemes through exhaustive testing using single and random selection of the row minima, as well as a more complex method of minimizing the sum of the remaining row minima, however, the end results were very similar. Therefore, *PaM* uses a single minimum selection for each row, and this selection is the minimum with the lowest column index *j.*

*PaM_full_* extends *PaM_simple_* by carrying out a more exhaustive pairing search. *PaM_full_* sorts the test cohort data iteratively in ascending order using the admixture components. The pairing procedure starts at a random row index (multiple times). The model starts by sorting the cohort data by the first admixture component then commences the search starting with a random row, *i*, index. The best pairing solution is stored. The cohort data is next sorted by the second admixture component, and the best pairing solution is found. If this solution minimizes the total GD of the final solution compared to the previous iteration then, the ‘sorted admixture component 2’ solution is stored. The model proceeds stepwise by successively sorting the remaining admixture components to find the best pairing solution. Poor pairs are handled in a similar manner to *PaM_simple_.* However, when the data are re-ordered, all previously discarded individuals are included in the new solution search.

### 2.5 Comparing *PaM* with demographic matches

*PaM* matches for the simulated datasets (Section 2.1) were compared with *a priori* matches based on age (within 5 years), gender and ‘race’ defined as ‘African’, ‘Asian’, ‘Latino’ or ‘White’. Following Elhaik *et al.* (2014) (Fig. 1), ancestry was inferred from the admixture components as follows: ‘African’ ancestry was assigned if the sum of Sub-Saharan Africa and South Africa admixture components was larger than 50%; ‘Asian’ ancestry was assigned when the North East Asian component was larger than 10% and ‘Latino’ ancestry was assigned when the Native American component was larger than 50%. All the remaining individuals were considered ‘White’. Since self-reported ‘race’ differs between studies, we considered three models: (i) an individual is either African, Asian, Latino or White. (ii) An individual is considered either an African or non-African; (iii) an individual is considered a mixture of Africans, Asians, Latinos and Whites. The assignment accuracy of all matches was measured based on the correct pairing of individuals and their known pairs with some individuals expected to be unpaired due to the removal of their exact match.

### 2.6 Comparing *PaM* with PCA matches

*PaM* matches for the Genographic dataset (Section 2.2) were compared with PCA-based methods. PCA’s top two eigenvectors were calculated using SNPRelate (Zheng *et al*., 2012). Clustering was achieved using the *k*-means method *kmeans* in *R.* Similar to Luca *et al.* (2008), pairs were determined by a random assignment within each cluster. To compare the quality of the results, the pairing solutions for *PaM* and PCA were evaluated using IBS clusters as an impartial GD independent of admixture or PCA and geographic distances calculated with the Haversine formula (Gellert *et al*., 1989). Due to the data’s high heterogeneity, *PaM* was employed with a threshold of 5.

### 2.7 Assessing *PaM’*s performances on small datasets

To evaluate *PaM*’s performances on small datasets (Section 2.2), we constructed four datasets consisting of 20, 40, 60 and 80 individuals. Each dataset consisted of an even number of Bedouins and Pakistanis. We applied *PaM_simple_* without a threshold to each dataset. To examine their effect, we also applied *PaM* with various thresholds (3, 5, and 7) to the largest dataset.

### 2.8 Comparing *PaM* with other clustering tools on unmixed and mixed individuals

*PaM* matches were compared with those of several clustering tools: PCA and multidimensional scaling (MDS), both available from PLINK, whose pairs were calculated as in Section 2.6; and genetic relationship matrix (GRM) (Yang *et al*., 2011) (version 1.91.2beta) and TreeMix (Pickrell and Pritchard, 2012) (version 1.13), whose pairs were identified using a greedy approach that paired individuals with the highest covariance. All the tools were assessed by their ability to match individuals in each dataset and reproduce the results in the combined datasets. The tools were applied to the complete and LD-pruned (PLINK command—indep 50 5 2) datasets and to the SNPs that overlapped *PaM*’s gene pools, which consist of ancestry informative markers (AIMs). *PaM* was utilized with three thresholds.

## 3 Results

### 3.1 Assessing *PaM*’s performances

We first evaluated the performances of *PaM_simple_* without a threshold (no limit placed on the acceptable score of pairs) and with a threshold of 7 (necessitating the matching of age/gender and at least six admixture components) across all simulated datasets. As expected, when applying *PaM* without a threshold, the GD increased with increasing perturbation or heterogeneity while the score decreased, and the number of misassigned pairs increased (Fig. 1, Supplementary Table S1). However, despite the perturbation and removal of individuals, most of the original pairs (80–100%) were correctly identified, particularly in Datasets 1–16. The increase in the number of misassigned pairs is related to how *PaM_simple_* searches for an optimum solution. *PaM_simple_* selects a case index *i* (row *i* of the GD matrix) and finds the best match for this index by locating the minimum GD in the row corresponding to the best possible match. This, however, does not constitute an ideal match considering all other individuals, some of whom are best left unpaired. Since *PaM_simple_* does not leave any individual unpaired (for an even cohort), a poor pairing may create a ‘snowball’ effect triggering other poor pairings, resulting in an overall increased GD and reduced score for the final pairing solution.

We addressed this problem by applying a threshold of 7 on the match score. Under these settings, the GD curve decreased sharply at a perturbation level of ∼5%; identifying genetically homogeneous pairs, despite the increased perturbation and discarding genetically mismatched pairs. The score, which represents the conservative choice of what is considered an acceptable pair, decreased due to the growing number of unpaired individuals. The trade-off for the low GD of acceptable pairs is that more individuals are left unpaired due to their low pairing score and are omitted from the total GD score (Fig. 1, Supplementary Table S1). The advantage of applying a threshold is that it reduces the number of misassigned pairs by allowing only pairs with a high match score (genetically homogeneous). This prevents the model from selecting pairs that satisfy the low GD minimum but do not have a favourable match score, thus avoiding the ‘snowball’ effect. Since the matrix is symmetric and each row has all possible pairing for each individual, individuals with a match score lower than the threshold are considered too genetically heterogeneous and placed on the unpaired list (Supplementary Table S2).

For Datasets 1–8, the best solution was obtained with a threshold at a perturbation level of 11%, where the GD was close to 0 for all matched pairs, nearly half the individuals had an acceptable score, and the number of misassigned pairs was 0. We note that for heavier perturbations, not all the misassignments are false positives since the perturbation created, by chance, more suitable pairing matches than the pre-defined ones. Considering the low GD between all pairs, the majority of matches were near-optimal ones even after removing individuals from the dataset. Interestingly, we observed a repeated single misassignment in most of the *remove-20* datasets (Supplementary Table S2). Examination of this unexpected misassignment showed it to be a pairing with a very low GD and a match score of 7, making it an acceptable assignment though not between the original partners, which could potentially be suboptimal.

There are two ways to address the vexing issue of ‘rogue’ misassignments. The first is to set a higher threshold, and the second is to use *PaM_full_*, which carries out a more exhaustive pairing search by iteratively sorting (in ascending order) the cohort data by the admixture components. The pairing procedure for the cohort commences at a random row index (multiple times). This approach does not produce rogue misassignments and hence finds an optimum or near-optimum pairing solution. The numerical results for the three datasets using *PaM_full_* are shown in Supplementary Tables S3 and S4. P*aM_full_* results are similar to those of *PaM_simple_*, except that they do not allow the accidental misassignments (Supplementary Table S4, perturbation <11%) observed with *PaM_simple_* (Supplementary Table S2).

As before, the misassignments detected beyond the 11% threshold are due to the high similarity in admixture components in the post-perturbation stage and are not truly false positives. The cost of using *PaM_full_* is increased computation time, almost an order of magnitude greater than *PaM_simple_*’s run time. Due to its superior performances, the remaining analyses were done with *PaM_simple_.*

### 3.2 Comparing the performances of *PaM* and alternative methods on simulated datasets

**Fig. 2.**
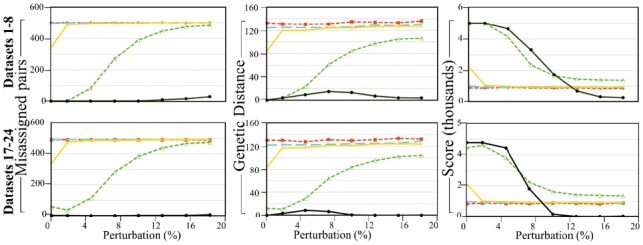
*PaM_simple_* (threshold of 7) performances on 16 simulated datasets against 5 competing methods. Columns show the number of misassigned individuals, total GD and pair score for Random assignment (red), Race model 1 (cyan), Race model 2 (yellow), Race model 3 (green) and *PaM* (black). Results for Datasets 9–16 were identical to those of Datasets 1–8 and are no shown

We next compared the assignment accuracy of *PaM_simple_* and alternative solutions in terms of misassigned pairs with the GD and Score illustrating the quality of the matches (Fig. 2). *PaM* correctly identified nearly all pairs. The GDs for the random assignment, where the age and gender matched but ‘race’ was randomly determined, were much larger than the competing solutions. Correspondingly, the random assignment’s score is mostly lower than the alternative solutions. Nearly none of the pairs randomly assigned were with their original counterparts.

The first two self-reported ‘race’ models (African, Asian, Latino or White; African/non-African) perform only slightly better than the random assignment in terms of GD and the score. The results of the third model (mixtures of African, Asian, Latino or White) are considerably better than the previous models or random assignments. This is to be expected, since this model can be considered a reduced form of *PaM*’s nine-admixture components model. Our results indicate that pairs obtained through standard demographics criteria (age, gender and self-defined ‘race’) are as poor as those obtained at random. We note, that since the simulated datasets comprised of the same admixture components used by *PaM,* and therefore, the performances observed in simulation may not reflect the algorithm’s accuracy for real populations.

### 3.3 Comparing *PaM*’s performances with PCA’s

We next compared the performances of *PaM* with a PCA-based approach on the Genographic dataset consisting of worldwide individuals alongside 13 simulated Indian–British individuals. *PaM* was applied to the admixture components of all individuals and PCA was applied to the SNP data.

**Fig. 3.**
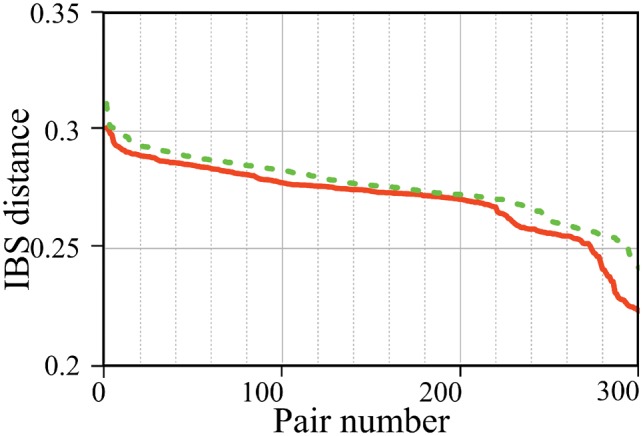
IBS distance between *PaM* (solid) and PCA (dashed) inferred pairs

**Fig. 4.**
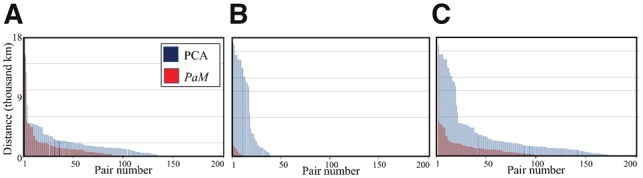
The geographical distance between individual pairs inferred by *PaM* and PCA. Geographic distances are calculated between pairs where both individuals are within the IBS-defined clusters (**A**), where individuals are in different clusters (**B**) and for all individuals regardless of cluster assignment (**C**)

We evaluated the homogeneity of the pairs inferred by *PaM* and PCA using both geographic and GDs. All of *PaM’s* inferred pairs had higher genetic similarity (i.e. smaller IBS distances) than PCA’s inferred pairs (Fig. 3). We identified 12 IBS clusters (Supplementary Fig. S4) and divided all inferred pairs to ‘matches’ if individuals were in the same cluster and ‘mismatches’ if otherwise. PCA pairs had 270 ‘matches’ and 46 ‘mismatches’ with mean distances of 1042 and 6124 km, respectively, and 10 unpaired individuals (Fig. 4). *PaM* pairs had 284 ‘matches’ and 17 ‘mismatches’ with mean distances of 484 and 557 km, respectively, and 40 unpaired individuals. Compared to PCA, individuals matched by *PaM* were significantly geographically closer regardless of the category (Kolmogorov–Smirnov goodness-of-fit test, *P-*value_(matches__)_ = 2.74*10 ^− ^^6^, *P-*value_(mismatches__)_ = 3.58*10 ^− ^^4^, *P-*value_(all__)_ = 4.85*10 ^− ^^10^). In one ‘match’ case, *PaM* paired an individual from Papua New Guinea with a Peruvian, which yielded a geographic distance of over 13 000 km. However, Skoglund *et al.* (2015) showed that some Native American populations can trace their origins to Papua New Guinea, suggesting that *PaM’s* assignment may have been appropriate. The 13 mixed Indo-British individuals formed a part of the Tartar/Tajikistan IBS cluster (Supplementary Fig. S4). PCA paired the Indo-British individuals with people from Tajikistan (4), Iran (2), Tatar (1), Russia (1), Ingush (1) and India (1). It correctly made one Indo-British pair and left out one individual. By contrast, *PaM* formed six Indo-British pairings, leaving the 13th individual unpaired (although the Indo-British were part of the same IBS cluster consisting of Tartars and Tajikistanians). Overall *PaM* produced pairs that are significantly more genetically (Fig. 3) and geographically (Fig. 4) homogeneous than PCA. These results highlight the accuracy of *PaM* and its ability to handle admixed individuals.

### 3.4 Evaluating *PaM*’s performances on small datasets

Applied to datasets ranging from 20 to 80 individuals of Bedouin and Pakistani descent (Supplementary Fig. S5), *PaM* (no threshold) perfectly paired all individuals with members of their populations each time. Applying *PaM* with higher thresholds to the largest dataset created slightly fewer pairs but more genetically homogeneous ones (Supplementary Table S5).

### 3.5 Comparing the performances of *PaM* and various clustering tools for unmixed and mixed individuals

**Fig. 5.**
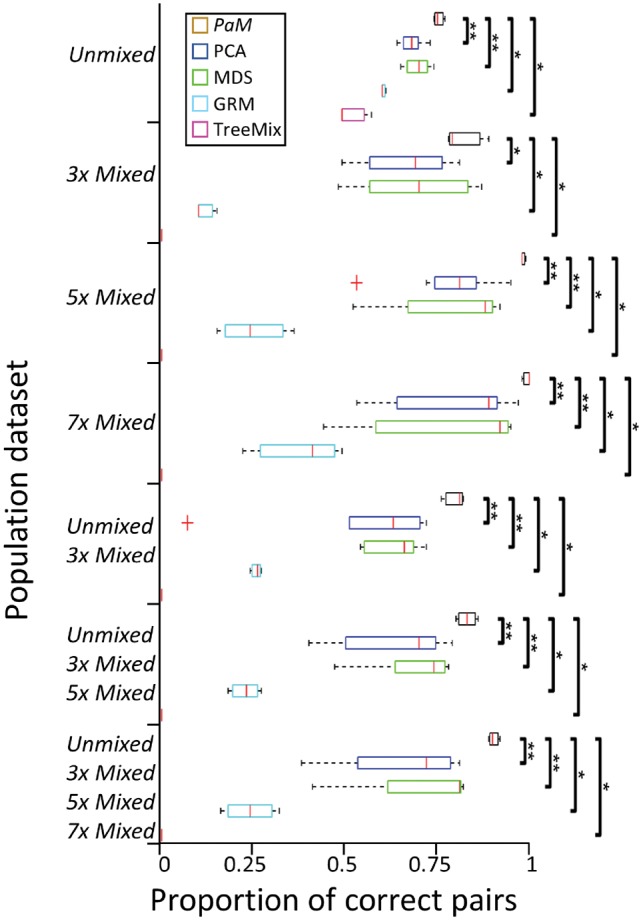
Pairing accuracy for various tools across multiple datasets. Boxplots summarize the pairing accuracy of all the trials in each population dataset (Supplementary Table S6), e.g. the PCA for unmixed individuals include the three analyses (PCA2/10/20) for each of the three datasets. The order of the tools’ results per population dataset is shown in the legend. Significance was estimated for *PaM* using Wilcoxon rank-sum test (*P*-value ≤ *0.05, ** ≤ 0.01)

Clustering accuracy is typically demonstrated by showing that well-curated individuals are predicted to geographic regions, whereas mixed individuals are more challenging to analyze under various population and data settings. Here, we evaluated the pairing accuracy of five tools that implement different clustering strategies in datasets that consist of unmixed and mixed individuals and combinations of those datasets. *PaM* significantly outperformed all tools in each test (Fig. 5, Supplementary Table S6), except in comparison to MDS in the 3 × Mixed dataset, with an average accuracy of 87 ± 9% compared to PCA (68 ± 16%), MDS (72 ± 14%), GRM (29 ± 16%) and TreeMix (7 ± 18%). The accuracy for PCA and MDS varied with the number of loadings used. The pairing with both 10 [76 ± 15% (PCA) and 79 ± 17% (MDS)] and 20 [71 ± 9% (PCA) and 78 ± 9% (MDS)] loadings was similar and higher than with two loadings [58 ± 10% (PCA) and 57 ± 10% (MDS)]. MDS outperformed PCA in nearly every test. TreeMix performed the worst. When admixed individuals were provided TreeMix reports were highly inaccurate (see a simplistic example in Supplementary Fig. S6). The combined datasets (unmixed + mixed), designed to test reproducibility, proved challenging, with *PaM* exhibiting the smallest drop in average accuracy (−5%), compared to PCA (−12%), MDS (−7%) and GRM (−9%). All tools performed better on the gene pool SNP set (59%) than on the LD-pruned (53%) and genome-wide datasets (57%).

### 3.6 Running time

Running on a single core Intel i5 computer, *PaM_simple_* finds the near-optimum pairings in ∼15 min for a test cohort of 1000 individuals, whereas *PaM_full_* finds the optimized pairings in ∼3 h. If accessed online, results are typically emailed within 20 min.

### 3.7 Software availability

*PaM* is freely available as a downloadable *R* package from https://github.com/eelhaik/PAM (16 Mb). In addition, a web-service has been created that allows users to upload genetic and demographic data for their test cohort in PLINK format and receive the optimized pairings solution by email (http://elhaik-matcher.sheffield.ac.uk/ElhaikLab/).

## 4 Discussion

Clinical trials are required to determine drug efficacy on multiple cohorts of sizes ranging from 40 to 10 000, where participants are split into treatment and control groups. The outcomes of these trials determine whether a drug should be tested in a larger cohort and, if successful, approved for use (Roy, 2012). To evaluate the therapeutic effects of the tested drug, treatment and control pairs have to be genetically homogeneous to minimize the variation in the response that is due to different genetic backgrounds. Therefore, pairing of cohort individuals is typically done at random after controlling for demographic criteria (e.g. age, sex and self-reported ‘race’) *a priori* to the trial. However, randomization does not resolve *population stratification*, particularly in very small cohorts or multiple strata with few individuals (Ganju and Zhou, 2011) and the results may not be replicated in a follow up larger trial, which may disqualify an effective drug. Correcting for population stratification *a posteriori* to the trial is also problematic due to the difficulty in modelling ancestry and admixture and the reliance on self-defined ‘race’, a highly unreliable predictor (De Bono, 1996; Fustinoni and Biller, 2000). A similar challenge exists in case-control genetic investigations intended to find a loci associated with a phenotype of interest. Unfortunately, even after decades of genetic research, the use of self-defined racial categorization is still highly prevalent in clinical settings. Though most of the genetic variation in humans is between continental populations (12%) (Elhaik, 2012) who exhibit biological variety, like different drug responses, racial terminology is an ineffective mean to classify mixed people, even those believed to be unmixed due to ignorance of their demographic history (e.g. Das *et al.*, 2017; Marshall *et al*., 2016).

Applying various tools to unmixed and mixed datasets provided a unique view of their clustering accuracy. We demonstrated that using standard demographic criteria, such as self-reported ‘race’ yields random results, suggesting that ancestry should be identified genetically (e.g. Baughn *et al*., 2018). We further showed that PCA pairings are geographically and genetically suboptimal and that it is incapable of modelling mixed populations (Figs 4 and 5), representing the vast majority of the population in countries like the USA. That PCA and PCA-like tools are still being used in GWAS and even considered the ‘gold standard’ by some and that PCA loadings from past GWAS are being used in GWAS meta-analyses is puzzling given PCA’s known weaknesses. The uneven sampling, for instance, which exists in any dataset biases PCA predictions (Elhaik *et al*., 2014; McVean, 2009). There is no consensus on the number of PCs to analyze: although Price *et al.* (2006) used a default of 10 PCs and Patterson *et al.* (2006) advised using the Tracy–Widom statistic to determine the number of components, in practic, authors use an arbitrary number of PCs or adopt *ad hoc* strategies to aid in their decision (e.g. Solovieff *et al*., 2010). This may be due to the high sensitivity of the Tracy–Widom statistics to linkage disequilibrium, which inflates the number of PCs (Patterson *et al.*, 2006) and the expectation that the PCs would reflect genetic similarities that are difficult to observe in higher PCs. PCA is also sensitive to the choice of markers (Supplementary Table S6). The GRM estimates the genetic relationship between two individuals and is one of the core functions of the GWAS package GCTA (Yang *et al*., 2011). It calculates the average ratio of the covariance over the expected heterogeneity across all genes. In other words, it represents how much two individuals covary relative to what is expected on average for an average SNP. This measure is susceptible to LD and cannot be expected to handle mixed individuals. Indeed, its best performances were for the unmixed individuals (Supplementary Table S6). Its prioritization over PCA (Yang *et al*., 2011) is, thereby, inconsistent with its low performances compared to PCA with two PCs. Remarkably, the less popular MDS outperformed PCA in almost every trial. This may be explained by the tendency of MDS to preserve pairwise distances between the points, which is in line with how the data were generated and evaluated. By contrast, PCA attempts to preserve the covariance of the data, which may be less sensitive to population structure. PCA’s requirement that the data will follow a multivariate normal distribution may also pose a challenge that does not exist in MDS. Our analysis of TreeMix results was based on the covariance matrix, which limitations were already discussed, rather than on the tree’s topology. This is because TreeMix’s furthest assumption that the history of the sampled populations is approximately tree-like (Pickrell and Pritchard, 2012) is not met in the mixed and combined datasets. Nonetheless, the limitations of the covariance matrix observed here (Supplementary Table S6) and TreeMix’s limitation in capturing complex admixture events (Lipson *et al*., 2013) are reflected in the poor performances of TreeMix (Supplementary Fig. S6). Interestingly, TreeMix also attempts to model migration, i.e. explain shared genetic similarity that cannot be properly modelled in a tree configuration. However, its predictions for humans (Pickrell and Pritchard, 2012) appear inconsistent, incomplete, and fit only partially with known population history. Phylogenetic models that represent admixture were proposed (Lipson *et al*., 2013); however, it is unclear how they can be applied to pair individuals. The dissonance between the commonality of these tools and their accuracy raises concerns that their popularity may be due to other factors than rigorousness and that these tools contribute to the reproducibility problem in clinical and medical studies.

As expected, all the methods performed better on the AIMs dataset than on the complete or LD-pruned ones as AIMs amplify the ancestral differences between individuals, whereas non-AIMs act to even the population differences. These findings imply that correcting for population structure will be more difficult in exome studies. In such studies investigators should utilize the few AIMs captured on their platform or genotype their samples on a dedicated population microarray (e.g. Elhaik *et al*., 2017).

Evaluated on simulated and real datasets, *PaM* outperformed all alternative classifiers. Among its advantages are high accuracy, a sample-independent approach that allows reproducibility (Fig. 5), and the use of a finite set of AIMs that improve the depiction of population structure (Supplementary Table S6). PaM’s admixture model has several more advantages. The admixture components are calculated relative to the putative ancestral populations so their meaning remains the same between different analyses. The admixture components allow intuitive and accurate insight into the ancestry (Supplementary Fig. S6) and geographical origins (Elhaik *et al*., 2014) of individuals. The genetic characterization of individuals can be used to identify subgroups of responders in drug trials, which can promote personalized medicine solutions tailored to population groups. To avoid suboptimal pairings when all pairs are assigned, we introduced a threshold for the minimum acceptable genetic similarity between tested pairs, which significantly reduced spurious assignments. The score and GD provided in the output allow further prioritization of the pairs. Though *PaM* seeks the best matching pair for each individual and is agnostic to the size of the dataset and admixture scheme, we caution from applying *PaM* to poorly constructed admixture schemes, which fail to capture the global genetic biodiversity. Finally, due to its short computational time, we recommend using *PaM_simple_* over *PaM_full_* the latter which performs a nearly exhaustive search.

In summary, we developed *PaM*—a software tool that employs demographic and genetic criteria to find optimized or near-optimized pairings solution for test cohorts consisting of unmixed and mixed individuals. *PaM* can be accessed online or be installed on the local computer.

## Supplementary Material

bty946_Supplementary_Data
