# Supplementary material for: Pair Matcher (PaM): fast model-based optimization of treatment/case-control matches
Source: Bioinformatics. 2018 Nov 16;35(13):2243–50. doi: 10.1093/bioinformatics/bty946 (PMC6596890; doi:10.1093/bioinformatics/bty946)
Supplement: bty946_Supplementary_Data [file bty946_supplementary_data.docx]

Supplementary Note

## Supplementary Figure 1

**Comparing the framework of a standard controlled trial and with *PaM* with a personalized medicine application.** A cohort of participants is available to study the effect of a new drug. Unknown to the tester, participants with a yellow allele, common among East Asians and rare elsewhere, respond better to the drug. In the standard framework, participants are divided at random to treatment and control groups and by average have a similar age, gender, and “race.” While the treatment appears to be successful in Phase II, the uneven distribution of the yellow allele in the Phase III trial deemed the treatment ineffective. *PaM* homogenizes the samples both *a priori* and *a* *posteriori* to the trial. After analysing *PaM*’s Phase II generated cohort the treatment is considered successful and after analyzing *PaM*’s records, it appears that East Asians are better responders. The Phase III results confirmed this findings allowing further development of a personalized medicine approach to treatment.


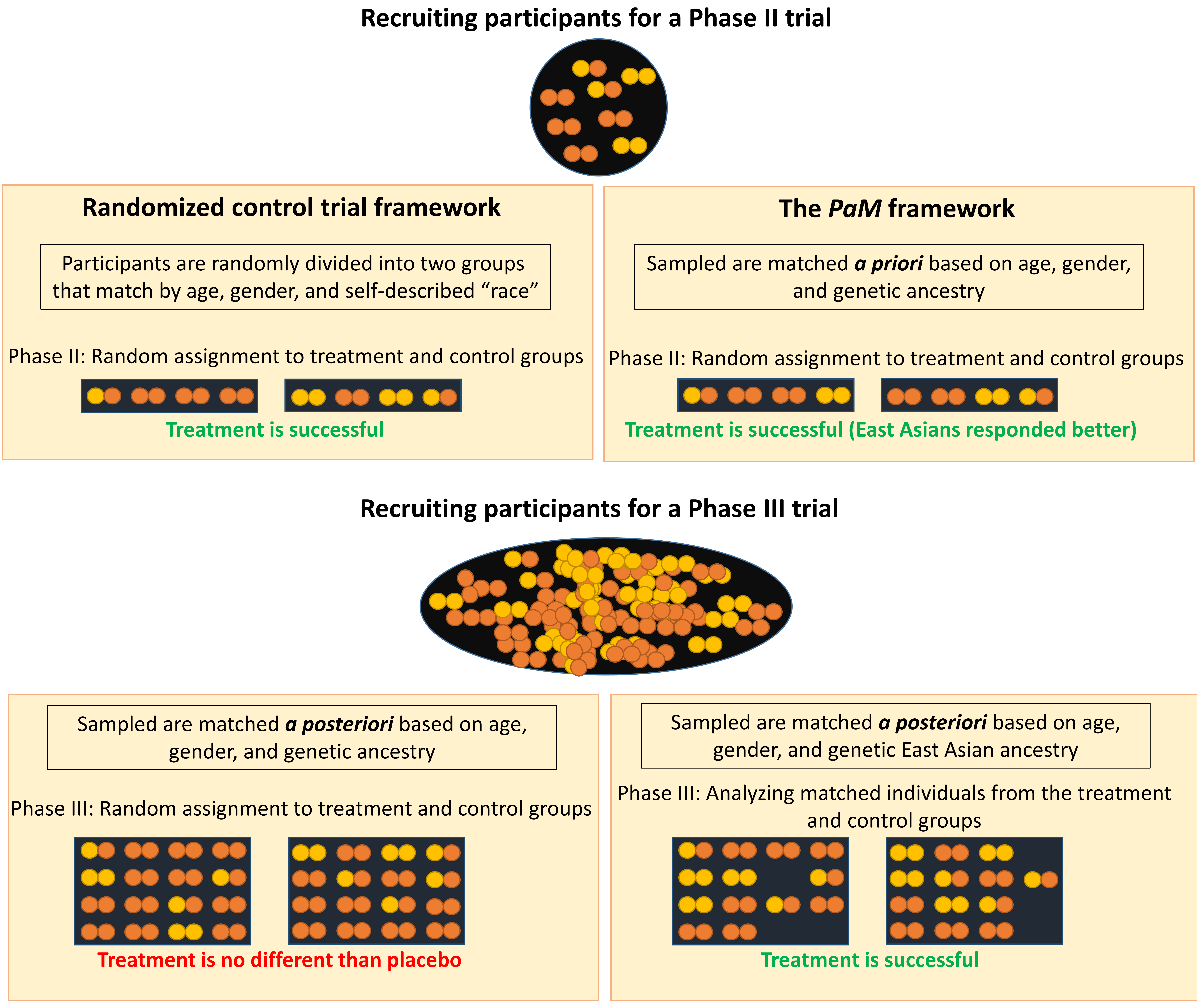


## Supplementary Figure 2

**The admixture components of ten random pairs, shown consecutively, from Datasets 1-8.** Each individual is represented by a vertical stacked column of colour-coded admixture components that reflects genetic contributions from the putative ancestral populations. The *x*-axis represents individual pairs from the unperturbed (0%) and perturbed (2-20%) datasets.


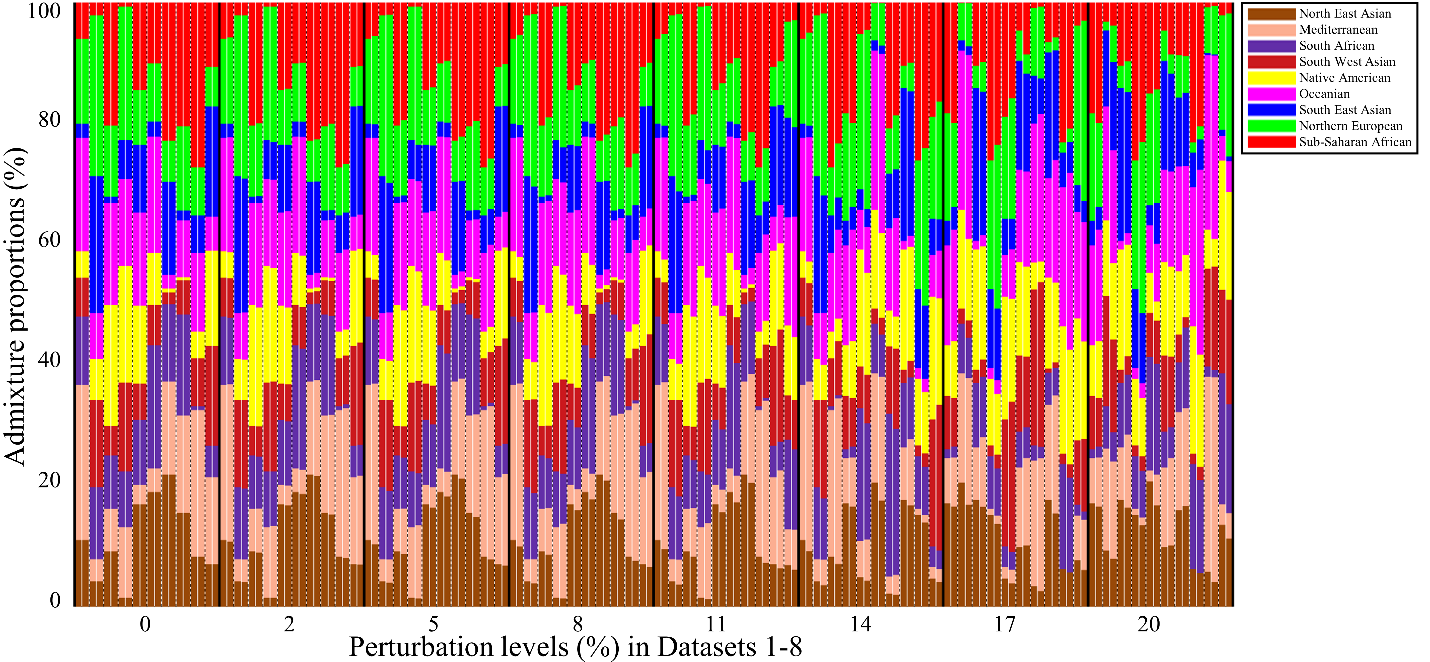


## Supplementary Figure 3

**An illustration on how pairing solutions are evaluated.** Consider an initial dataset with six genetically matched pairs. One individual was then randomly removed. An attempt was made to match the pairs using *PaM_simple_* that connected 3-4 and *PaM_full_*. *PaM*’s pairing solutions with and without threshold are evaluated based on five criteria. The results differ based on whether or not a threshold was applied.
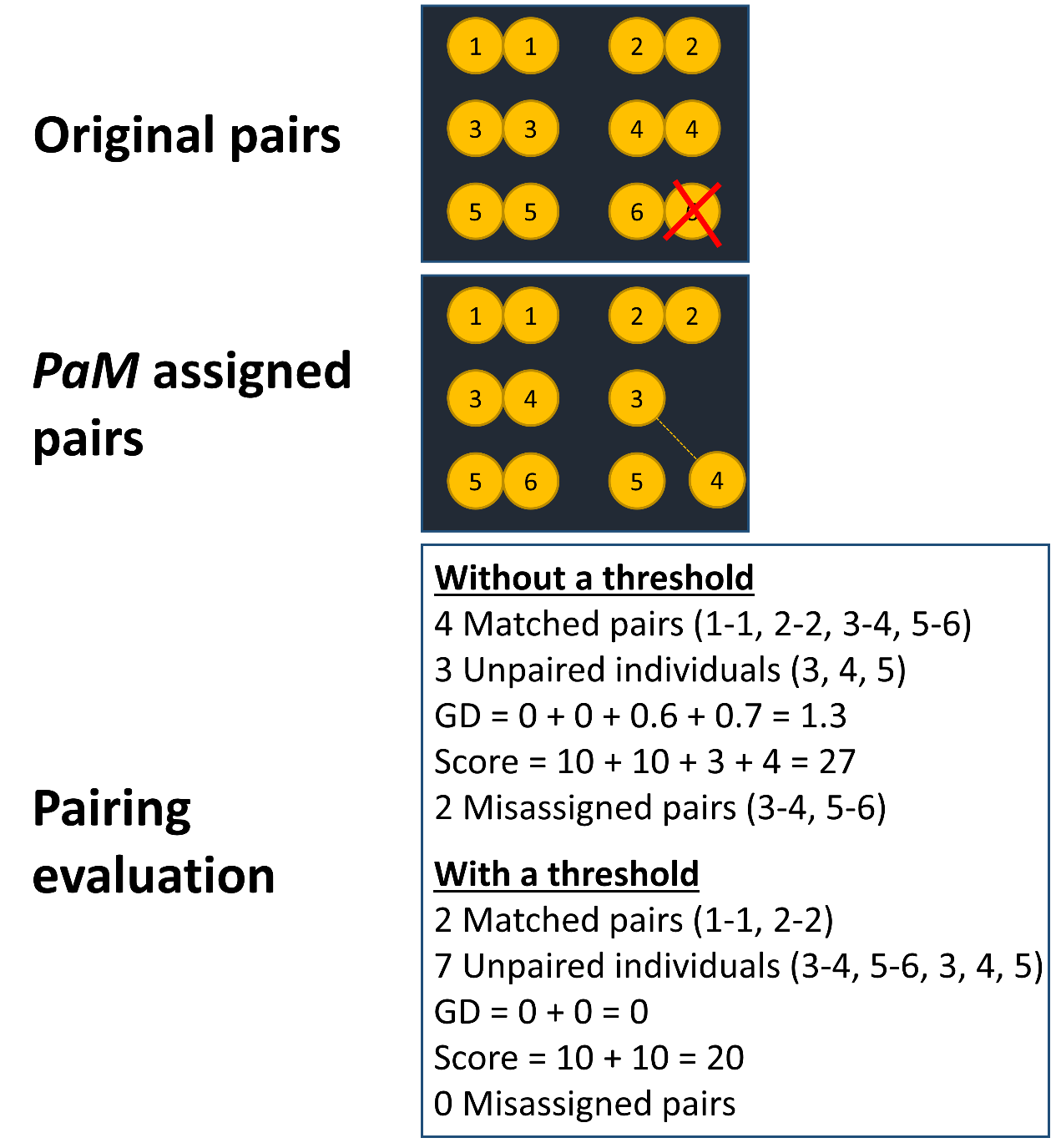


## Supplementary Figure 4

**PCA plot of genetic diversity across the worldwide populations**. The figure represents the genetic diversity seen across the populations considered, mapped onto a spectrum of genetic variation represented by two axes of variations corresponding to two eigenvectors of the PCA. Individuals from each population cluster are marked by region that are represented by unique shape and color.


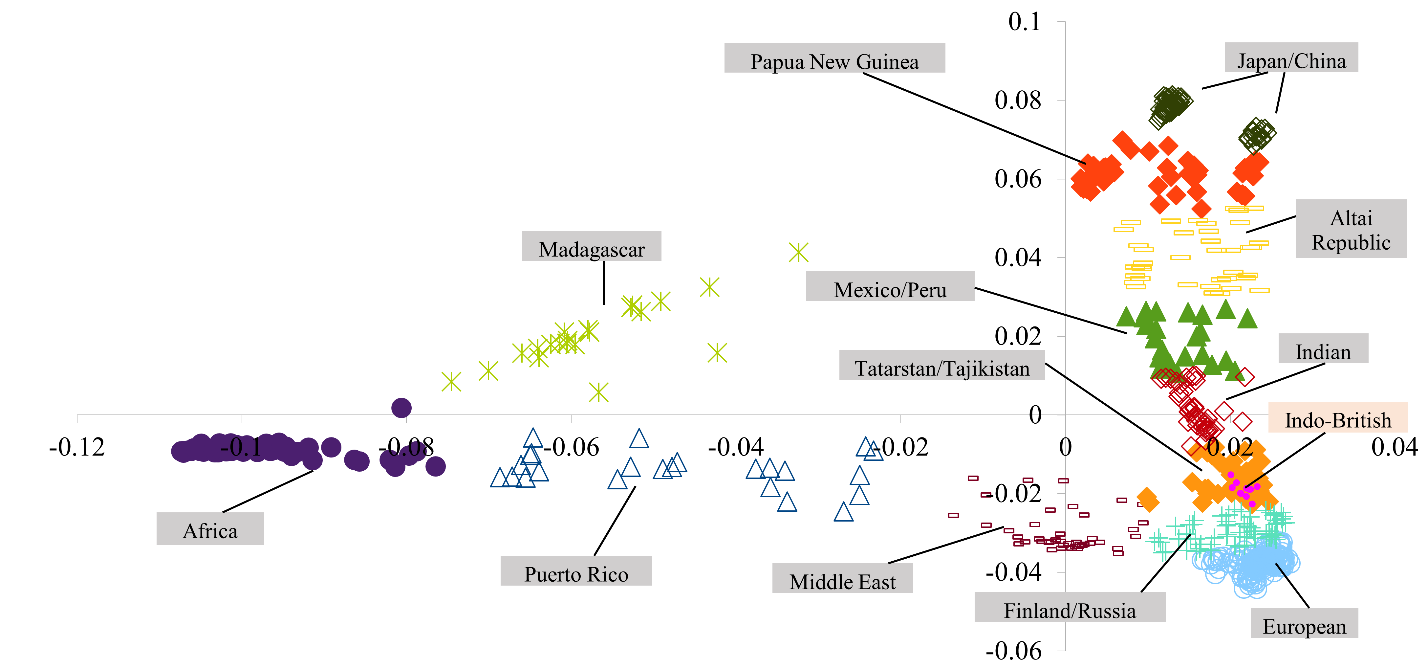


## Supplementary Figure 5

**The admixture components of 40 Bedouin and 40 Pakistani individuals (25 Brahui and 15 Burusho).** Each individual is represented by a vertical stacked column of colour-coded admixture components that reflects genetic contributions from the putative ancestral populations.


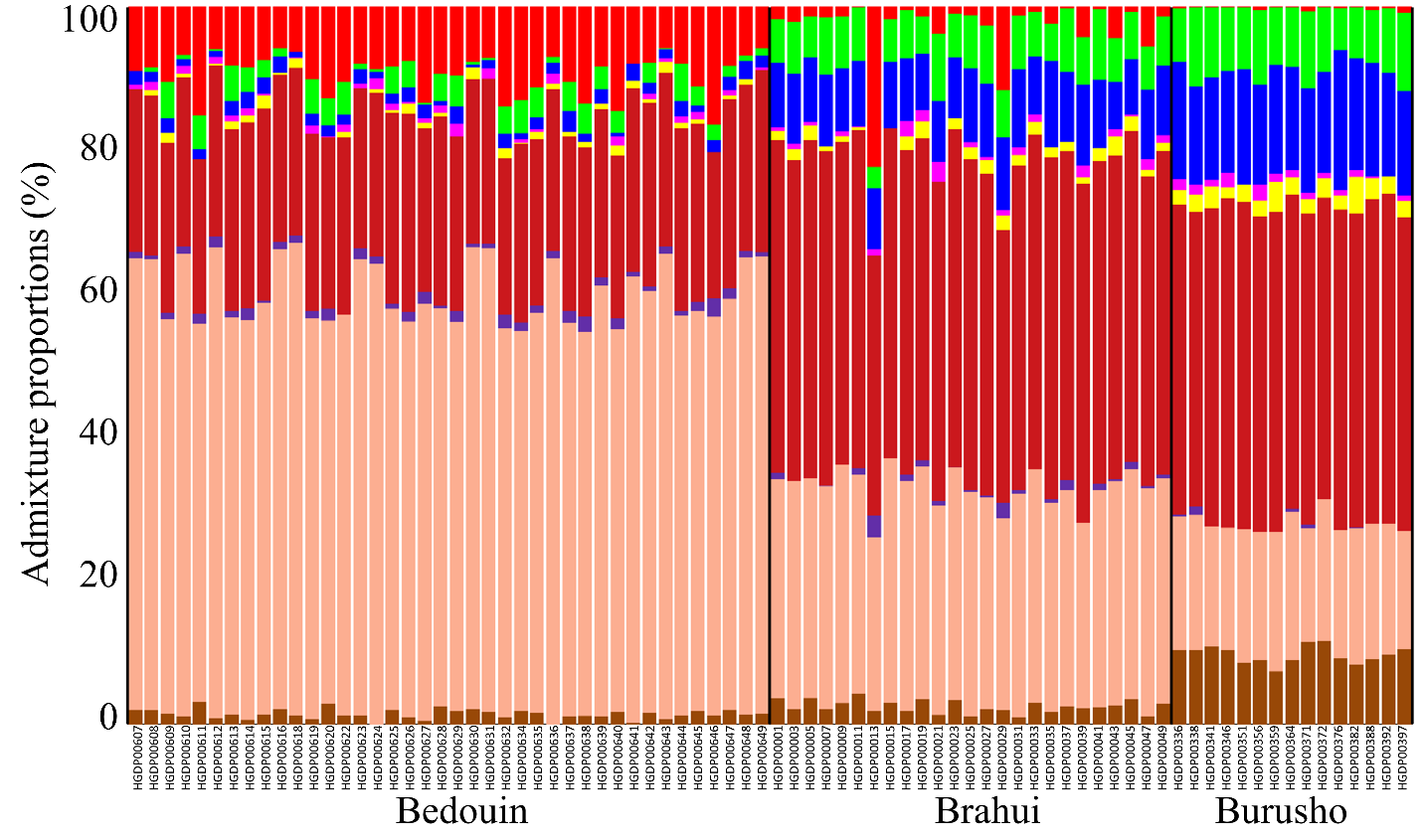


## Supplementary Figure 6


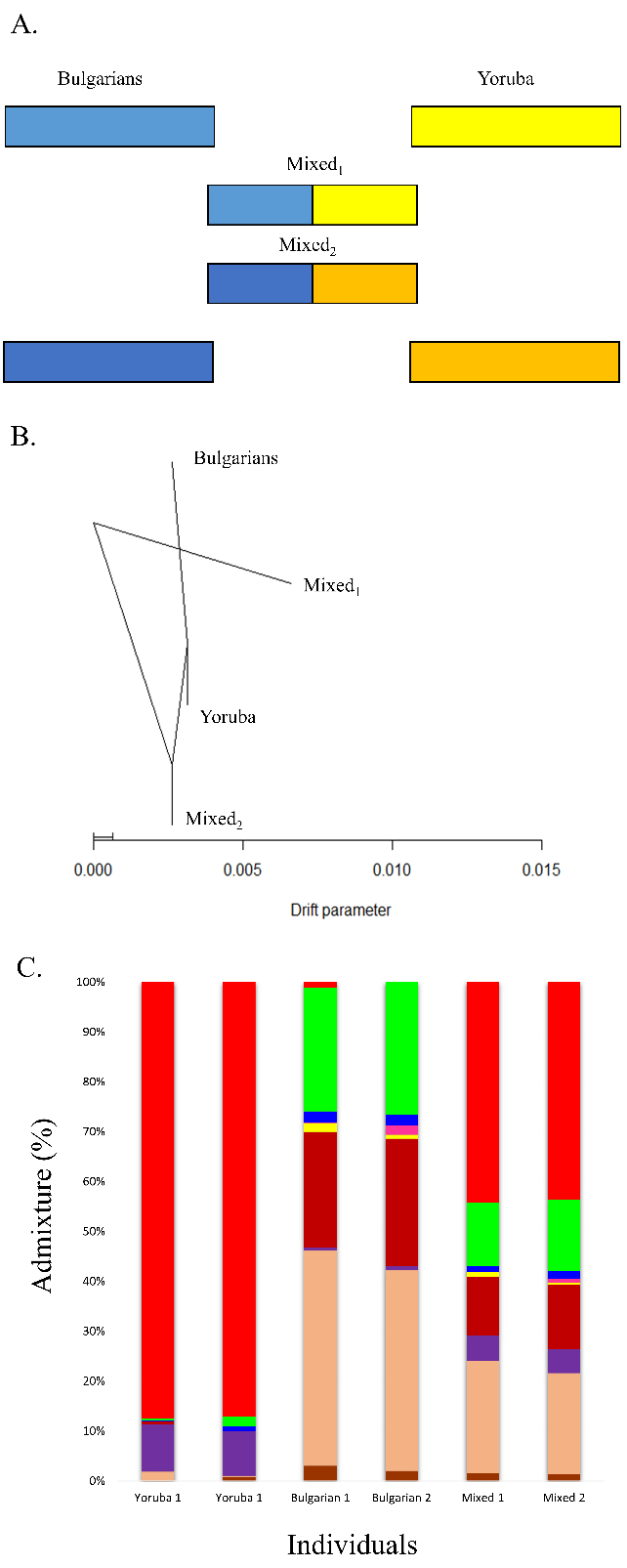
**TreeMix and *PaM* applications to unmixed and mixed individuals.** Two Yoruba and two Bulgarians were randomly selected from the Lazaridis et al. (2014) dataset. Two mixed individuals were created by copying half of the genotypes of the founders as in A). TreeMix results (B) and PaM’s admixture components (C) for these genomes are shown.

## Supplementary Table 1

***PaM_simple_* results without a threshold**. Results are shown per dataset type and level of perturbation. The number of matched pairs are shown along with their total GD, total score, and the number of misassigned pairs. The remaining unpaired individuals are shown last.

|  | Perturbation (%) | Matched pairs | | | | Number of unpaired individuals |
| --- | --- | --- | --- | --- | --- | --- |
| Full cohort |  | Number | Total GD | Total score | Misassigned |  |
|  | 0 | 500 | 0 | 5000 | 0 | 0 |
|  | 2 | 500 | 3.67 | 5000 | 0 | 0 |
|  | 5 | 500 | 9.13 | 4640 | 0 | 0 |
|  | 8 | 500 | 14.65 | 3319 | 0 | 0 |
|  | 11 | 500 | 20.21 | 2434 | 0 | 0 |
|  | 14 | 500 | 25.78 | 1952 | 6 | 0 |
|  | 17 | 500 | 31.91 | 1652 | 36 | 0 |
|  | 20 | 500 | 37.98 | 1496 | 73 | 0 |
| *Remove-1* | 0 | 499 | 0.102 | 4984 | 1 | 1 |
|  | 2 | 499 | 3.756 | 4984 | 1 | 1 |
|  | 5 | 499 | 9.442 | 4604 | 4 | 1 |
|  | 8 | 499 | 14.906 | 3300 | 4 | 1 |
|  | 11 | 499 | 20.326 | 2424 | 3 | 1 |
|  | 14 | 499 | 25.853 | 1941 | 9 | 1 |
|  | 17 | 499 | 32.096 | 1648 | 38 | 1 |
|  | 20 | 499 | 37.672 | 1496 | 64 | 1 |
| *Remove-20* | 0 | 490 | 3.778 | 4695 | 29 | 0 |
|  | 2 | 490 | 7.504 | 4663 | 33 | 0 |
|  | 5 | 490 | 12.888 | 4306 | 37 | 0 |
|  | 8 | 490 | 17.979 | 3119 | 39 | 0 |
|  | 11 | 490 | 22.652 | 2312 | 35 | 0 |
|  | 14 | 490 | 27.712 | 1874 | 38 | 0 |
|  | 17 | 490 | 33.622 | 1598 | 63 | 0 |
|  | 20 | 490 | 38.721 | 1452 | 99 | 0 |

## Supplementary Table 2

***PaM_simple_* results with a threshold of 7**. Results are shown per dataset type and level of perturbation. The number of matched pairs are shown along with their total GD, total score, and the number of misassigned pairs. The remaining unpaired individuals are shown last.

|  | Perturbation (%) | Matched pairs | | | | Number of unpaired individuals |
| --- | --- | --- | --- | --- | --- | --- |
|  |  | Number | Total GD | Total score | Misassigned |  |
| Full cohort | 0 | 500 | 0 | 5000 | 0 | 0 |
|  | 2 | 500 | 3.666 | 5000 | 0 | 0 |
|  | 5 | 500 | 9.125 | 4640 | 0 | 0 |
|  | 8 | 248 | 7.081 | 1867 | 0 | 504 |
|  | 11 | 24 | 0.855 | 176 | 0 | 952 |
|  | 14 | 5 | 0.225 | 35 | 2 | 990 |
|  | 17 | 4 | 0.191 | 28 | 3 | 992 |
|  | 20 | 7 | 0.407 | 49 | 7 | 986 |
| *Remove-1* | 0 | 499 | 0 | 4990 | 0 | 1 |
|  | 2 | 499 | 3.659 | 4990 | 0 | 1 |
|  | 5 | 499 | 9.109 | 4630 | 0 | 1 |
|  | 8 | 248 | 7.081 | 1867 | 0 | 503 |
|  | 11 | 24 | 0.855 | 176 | 0 | 951 |
|  | 14 | 5 | 0.225 | 35 | 2 | 989 |
|  | 17 | 4 | 0.191 | 28 | 3 | 991 |
|  | 20 | 7 | 0.407 | 49 | 7 | 985 |
| *Remove-20* | 0 | 480 | 0.049 | 4797 | 1 | 20 |
|  | 2 | 480 | 3.554 | 4797 | 1 | 20 |
|  | 5 | 480 | 8.756 | 4454 | 0 | 20 |
|  | 8 | 241 | 6.887 | 1817 | 1 | 498 |
|  | 11 | 24 | 0.859 | 176 | 1 | 932 |
|  | 14 | 4 | 0.171 | 28 | 1 | 972 |
|  | 17 | 3 | 0.137 | 21 | 2 | 974 |
|  | 20 | 6 | 0.352 | 42 | 6 | 968 |

## Supplementary Table 3

***PaM_full_* results without a threshold**. Results are shown per dataset type and level of perturbation. The number of matched pairs are shown along with their total GD, total score, and the number of misassigned pairs. The remaining unpaired individuals are shown last.

|  | Perturbation (%) | Matched pairs | | | | Number of unpaired individuals |
| --- | --- | --- | --- | --- | --- | --- |
|  |  | Number | Total GD | Total score | Misassigned |  |
| Full cohort | 0 | 500 | 0 | 5000 | 0 | 0 |
|  | 2 | 500 | 3.666 | 5000 | 0 | 0 |
|  | 5 | 500 | 9.125 | 4640 | 0 | 0 |
|  | 8 | 500 | 14.645 | 3319 | 0 | 0 |
|  | 11 | 500 | 20.207 | 2434 | 0 | 0 |
|  | 14 | 500 | 25.781 | 1952 | 6 | 0 |
|  | 17 | 500 | 31.416 | 1633 | 21 | 0 |
|  | 20 | 500 | 37.023 | 1504 | 75 | 0 |
| *Remove-1* | 0 | 499 | 0.302 | 4978 | 2 | 1 |
|  | 2 | 499 | 3.991 | 4977 | 2 | 1 |
|  | 5 | 499 | 9.331 | 4619 | 2 | 1 |
|  | 8 | 499 | 14.809 | 3307 | 2 | 1 |
|  | 11 | 499 | 20.171 | 2429 | 0 | 1 |
|  | 14 | 499 | 25.74 | 1939 | 4 | 1 |
|  | 17 | 499 | 32.046 | 1630 | 33 | 1 |
|  | 20 | 499 | 37.427 | 1511 | 80 | 1 |
| *Remove-20* | 0 | 490 | 5.998 | 4555 | 50 | 0 |
|  | 2 | 490 | 8.482 | 4614 | 42 | 0 |
|  | 5 | 490 | 13.808 | 4261 | 45 | 0 |
|  | 8 | 490 | 17.948 | 3098 | 42 | 0 |
|  | 11 | 490 | 23.032 | 2309 | 38 | 0 |
|  | 14 | 490 | 27.852 | 1886 | 39 | 0 |
|  | 17 | 490 | 33.44 | 1598 | 67 | 0 |
|  | 20 | 490 | 38.441 | 1498 | 127 | 0 |

## Supplementary Table 4

***PaM_full_* results with a threshold of 7**. Results are shown per dataset type and level of perturbation. The number of matched pairs are shown along with their total GD, total score, and the number of misassigned pairs. The remaining unpaired individuals are shown last.

|  | Perturbation (%) | Matched pairs | | | | Number of unpaired individuals |
| --- | --- | --- | --- | --- | --- | --- |
|  |  | Number | Total GD | Total score | Misassigned |  |
| Full cohort | 0 | 500 | 0 | 5000 | 0 | 0 |
|  | 2 | 500 | 3.666 | 5000 | 0 | 0 |
|  | 5 | 500 | 9.125 | 4640 | 0 | 0 |
|  | 8 | 248 | 7.081 | 1867 | 0 | 504 |
|  | 11 | 24 | 0.855 | 176 | 0 | 952 |
|  | 14 | 5 | 0.225 | 35 | 2 | 990 |
|  | 17 | 4 | 0.191 | 28 | 3 | 992 |
|  | 20 | 7 | 0.407 | 49 | 7 | 986 |
| *Remove-1* | 0 | 499 | 0 | 4990 | 0 | 1 |
|  | 2 | 499 | 3.659 | 4990 | 0 | 1 |
|  | 5 | 499 | 9.109 | 4630 | 0 | 1 |
|  | 8 | 248 | 7.081 | 1867 | 0 | 503 |
|  | 11 | 24 | 0.855 | 176 | 0 | 951 |
|  | 14 | 5 | 0.225 | 35 | 2 | 989 |
|  | 17 | 4 | 0.191 | 28 | 3 | 991 |
|  | 20 | 7 | 0.407 | 49 | 7 | 985 |
| *Remove-20* | 0 | 480 | 0 | 4800 | 0 | 20 |
|  | 2 | 480 | 3.515 | 4800 | 0 | 20 |
|  | 5 | 480 | 8.756 | 4454 | 0 | 20 |
|  | 8 | 240 | 6.838 | 1810 | 0 | 500 |
|  | 11 | 24 | 0.859 | 176 | 1 | 932 |
|  | 14 | 4 | 0.171 | 28 | 1 | 972 |
|  | 17 | 3 | 0.137 | 21 | 2 | 974 |
|  | 20 | 6 | 0.352 | 42 | 6 | 968 |

## Supplementary Table 5

***PaM_simple_* results for a cohort of 40 Bedouin and 40 Pakistani individuals (25 Brahui and 15 Burusho) with various thresholds**. Results demonstrated the tradeoff in applying low and high thresholds with low thresholds maximizing the number of pairs and high threshold maximizing the genetic homogeneity between the pairs and leaving a growing number of individuals unpaired.

| Threshold | 1 | 3 | 5 | 7 |
| --- | --- | --- | --- | --- |
| Bedouin-Bedouin matches | 40 | 40 | 34 | 32 |
| Pakistani-Pakistani matches | 40 | 40 | 36 | 22 |
| Unpaired Bedouin | 0 | 0 | 6 | 8 |
| Unpaired Pakistani | 0 | 0 | 4 | 18 |
| Total individuals | 80 | 80 | 80 | 80 |
| Mean Score | 6.4 | 6.4 | 7.1 | 8.0 |
| Mean GD | 0.04 | 0.04 | 0.03 | 0.02 |

## Supplementary Table 6

**Comparing the pairing accuracy of multiple tools for unmixed and mixed individuals across 21 SNP and population datasets.** The three SNP sets (extended rows) vary by the number of SNPs. The seven population sets (columns) vary by the type and number of individuals. Five tools in various settings (rows) were applied to each dataset and the pairing accuracy is shown. The loading number used for PCA and MDS is noted in parenthesis. *PaM_simple_* was applicable only to the first dataset and was executed with various thresholds.

| Dataset | Tool | Unmixed  (*n*=200) | 3x Mixed  (*n*=200) | 5x Mixed  (*n*=200) | 7x Mixed  (*n*=200) | Unmixed 3x Mixed  (*n*=400) | Unmixed 3x Mixed  5x Mixed  (*n*=600) | Unmixed 3x Mixed  5x Mixed  7x Mixed  (*n*=800) |
| --- | --- | --- | --- | --- | --- | --- | --- | --- |
| Gene pools  (50,706 SNPs) | *PaM (3)* | 0.75 | 0.79 | 0.98 | 0.98 | 0.81 | 0.80 | 0.89 |
|  | *PaM (5)* | 0.74 | 0.78 | 0.99 | 1 | 0.82 | 0.86 | 0.92 |
|  | *PaM (7)* | 0.77 | 0.89 | 0.98 | 1 | 0.76 | 0.83 | 0.9 |
|  | PCA (2) | 0.65 | 0.59 | 0.75 | 0.65 | 0.51 | 0.51 | 0.51 |
|  | PCA (10) | 0.68 | 0.81 | 0.95 | 0.97 | 0.71 | 0.79 | 0.81 |
|  | PCA (20) | 0.72 | 0.72 | 0.84 | 0.90 | 0.66 | 0.76 | 0.80 |
|  | MDS (2) | 0.65 | 0.57 | 0.67 | 0.56 | 0.54 | 0.56 | 0.54 |
|  | MDS (10) | 0.70 | 0.84 | 0.92 | 0.95 | 0.68 | 0.78 | 0.81 |
|  | MDS (20) | 0.73 | 0.70 | 0.88 | 0.92 | 0.67 | 0.77 | 0.82 |
|  | GRM | 0.60 | 0.15 | 0.36 | 0.49 | 0.27 | 0.27 | 0.32 |
|  | TreeMix | 0.57 | 0 | 0 | 0 | 0 | 0 | 0 |
| Whole data  (600,841 SNPs) | PCA (2) | 0.69 | 0.55 | 0.72 | 0.61 | 0.56 | 0.50 | 0.54 |
|  | PCA (10) | 0.68 | 0.80 | 0.90 | 0.95 | 0.72 | 0.74 | 0.78 |
|  | PCA (20) | 0.73 | 0.69 | 0.81 | 0.90 | 0.07 | 0.70 | 0.74 |
|  | MDS (2) | 0.67 | 0.55 | 0.67 | 0.59 | 0.55 | 0.66 | 0.64 |
|  | MDS (10) | 0.72 | 0.87 | 0.90 | 0.95 | 0.72 | 0.77 | 0.82 |
|  | MDS (20) | 0.69 | 0.78 | 0.90 | 0.92 | 0.66 | 0.75 | 0.81 |
|  | GRM | 0.61 | 0.10 | 0.24 | 0.41 | 0.26 | 0.23 | 0.24 |
|  | TreeMix | 0.49 | 0 | 0 | 0 | 0 | 0 | 0 |
| LD pruned (167,904 SNPs) | PCA (2) | 0.64 | 0.49 | 0.53 | 0.53 | 0.51 | 0.50 | 0.54 |
|  | PCA (10) | 0.66 | 0.75 | 0.82 | 0.89 | 0.70 | 0.40 | 0.38 |
|  | PCA (20) | 0.67 | 0.57 | 0.78 | 0.84 | 0.63 | 0.70 | 0.72 |
|  | MDS (2) | 0.66 | 0.48 | 0.52 | 0.44 | 0.55 | 0.47 | 0.41 |
|  | MDS (10) | 0.70 | 0.83 | 0.88 | 0.94 | 0.70 | 0.74 | 0.81 |
|  | MDS (20) | 0.74 | 0.65 | 0.84 | 0.93 | 0.65 | 0.73 | 0.79 |
|  | GRM | 0.60 | 0.10 | 0.15 | 0.22 | 0.24 | 0.18 | 0.16 |
|  | TreeMix | 0.49 | 0 | 0 | 0 | 0 | 0 | 0 |

## References

Lazaridis I, et al. 2014. Ancient human genomes suggest three ancestral populations for present-day Europeans. Nature. 513:409-413.
